# Ulcerated Gastric Lipoma Presenting with Gastrointestinal Bleeding and Hypovolemic Shock

**DOI:** 10.5334/jbsr.2700

**Published:** 2022-01-17

**Authors:** Pedro Marques, Alexandra Santos, Ana Germano

**Affiliations:** 1 Hospital Prof. Doutor Fernando Fonseca, PT

**Keywords:** gastric lipoma, gastrointestinal bleeding, CT

## Abstract

**Teaching Point:** Gastric lipomas are very rare and generally asymptomatic benign tumors; however, they can manifest as life-threatening complications, readily diagnosed by CT.

## Case History

**Figure 1:**
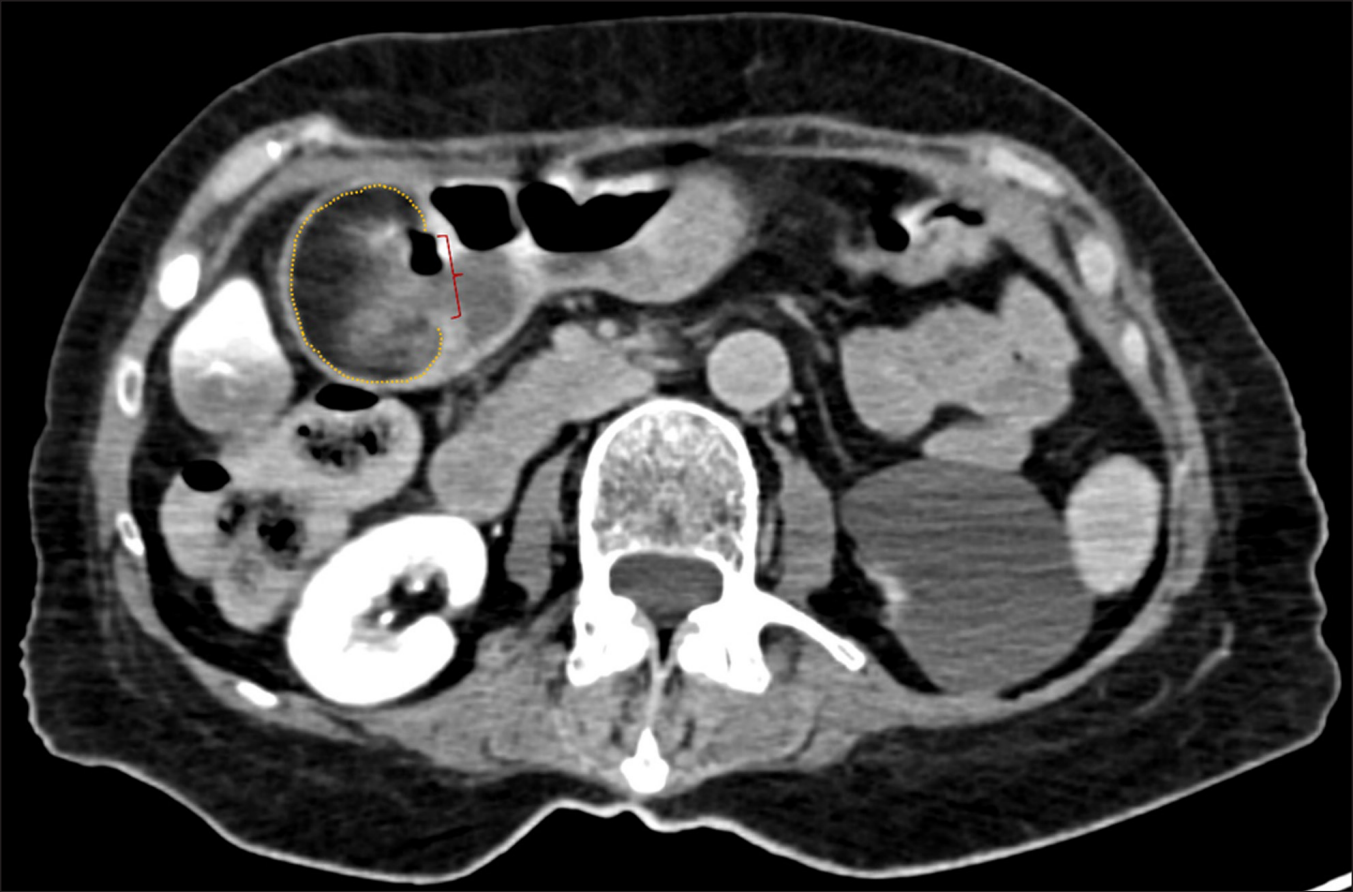


**Figure 2:**
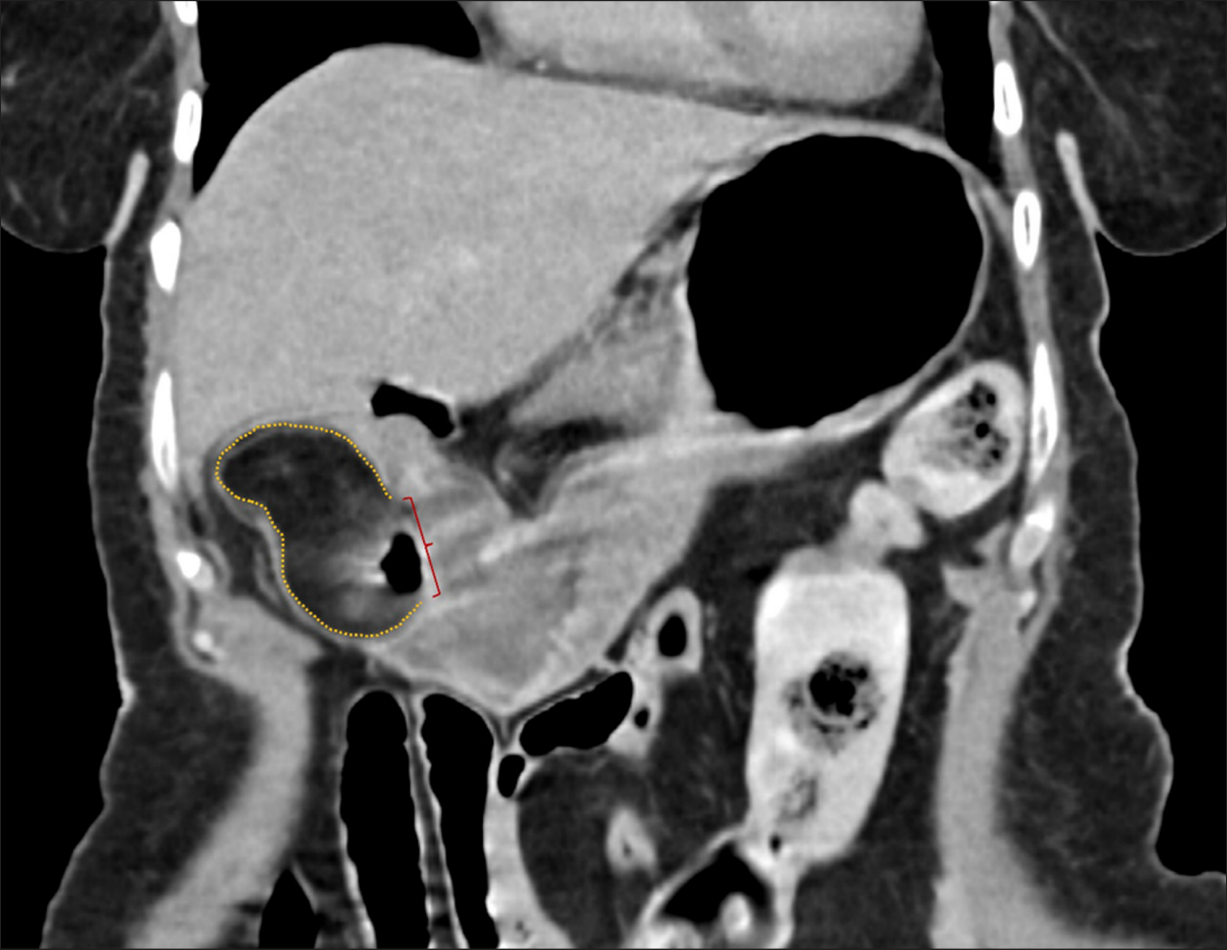


**Figure 3:**
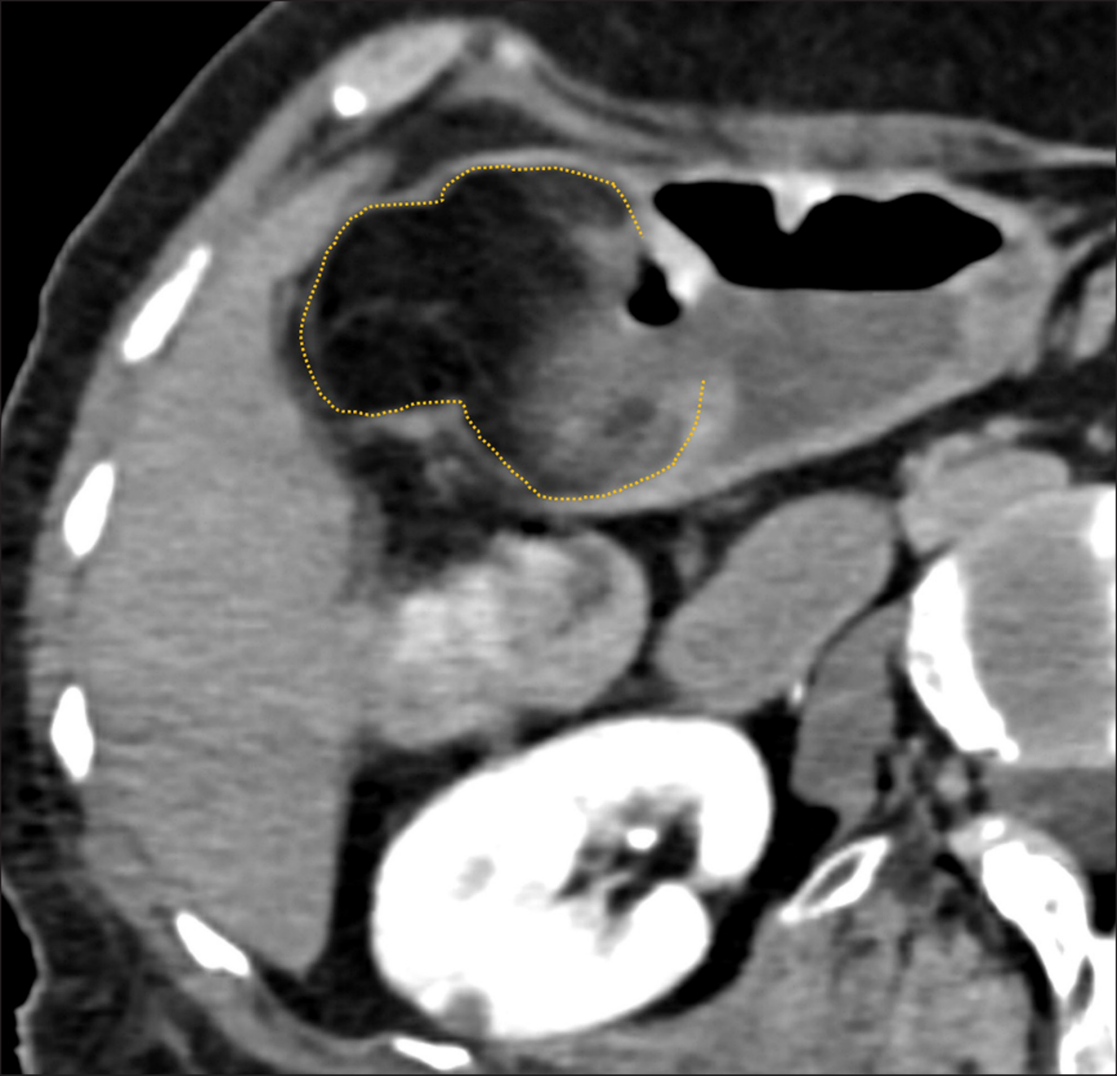


A 73-year-old female was brought to our emergency department for being found by her son in bed, prostrated, cold, and not reactive. Patient’s relevant medical history was depression and dementia syndrome. Physical examination revealed Glasgow coma scale of 9, paleness, cyanosis, hypothermia, hypotension (50/20 mmHg), and tachycardia (109 bpm). Digital rectal examination without evidence of active bleeding. Analytically, peripheral oxygen saturation of 85%, low hemoglobin (5,9 g/dL), leukocytosis (29 × 10^9^/L), high C-reactive protein (5,95 mg/L), and high creatinine (5,17 mg/dL) were found. Hypovolemic shock of undetermined origin was assumed, and fluid therapy with warm saline was started. Due to the impossibility of providing a history, the patient underwent head computed tomography (CT) with intravenous contrast and chest radiograph, both without significant abnormalities. After stabilization, a non-contrast-enhanced (due to renal function worsening) abdominopelvic CT was performed, which revealed a bulky oval image of fat density in the gastric antrum wall, apparently submucosal, measuring 60 × 44 × 41 mm, suggestive of a gastric lipoma (dotted line on axial, ***Figure 1***; coronal, ***Figure 2***; and oblique-axial, ***Figure 3***, planes). A solution of continuity was observed in the lesion’s inferior aspect, compatible with an ulcerated area (bracket on ***Figures 1*** and ***2***), containing near air and high-density areas. These findings were consistent with an ulcerated submucosal fat-containing lesion of the gastric antrum, with probable bleeding. The patient underwent subtotal gastrectomy with gastro-jejunal anastomosis. The specimen histological evaluation confirmed a gastric lipoma with wide steatonecrosis, located in the submucosal and muscular layers and extending to the mucosa with an area of ulceration. No evidence of atypia was found. Thus, the diagnostic hypothesis of hypovolemic shock caused by upper gastrointestinal (GI) bleeding originating from an ulcerated gastric lipoma was confirmed.

## Comment

Gastric lipomas are very rare tumors, corresponding to less than 3% of benign gastric tumors and less than 5% of all gastrointestinal lipomas [1]. Gastric lipomas are usually solitary tumors, located in the submucosa of the antrum. Most are asymptomatic and their diagnosis is incidental [1]. Rarely, larger lipomas (>3 cm) can become symptomatic, manifesting both by mild and chronic symptoms (abdominal pain and slight GI bleeding due to ulceration) or by serious and potentially fatal complications, such as gastric outlet obstruction, gastroduodenal intussusception and massive acute GI bleeding. In these last situations, all of which can be diagnosed by CT, surgical excision of the lipoma is recommended [1].
